# Texture Analysis of Fat-Suppressed T2-Weighted Magnetic Resonance Imaging and Use of Machine Learning to Discriminate Nasal and Paranasal Sinus Small Round Malignant Cell Tumors

**DOI:** 10.3389/fonc.2021.701289

**Published:** 2021-12-13

**Authors:** Chen Chen, Yuhui Qin, Junying Cheng, Fabao Gao, Xiaoyue Zhou

**Affiliations:** ^1^ Department of Radiology, West China Hospital, Sichuan University, Chengdu, China; ^2^ Department of MRI, The First Affiliated Hospital of Zhengzhou University, Zhengzhou, China; ^3^ MR Collaboration, Siemens Healthineers Ltd., Shanghai, China

**Keywords:** Fs-T2WI, artificial intelligence, machine learning, texture analysis, radiomics, small round cell malignant tumors

## Abstract

**Objective:**

We used texture analysis and machine learning (ML) to classify small round cell malignant tumors (SRCMTs) and Non-SRCMTs of nasal and paranasal sinus on fat-suppressed T2 weighted imaging (Fs-T2WI).

**Materials:**

Preoperative MRI scans of 164 patients from 1 January 2018 to 1 January 2021 diagnosed with SRCMTs and Non-SRCMTs were included in this study. A total of 271 features were extracted from each regions of interest. Datasets were randomly divided into two sets, including a training set (∼70%) and a test set (∼30%). The Pearson correlation coefficient (PCC) and principal component analysis (PCA) methods were performed to reduce dimensions, and the Analysis of Variance (ANOVA), Kruskal-Wallis (KW), and Recursive Feature Elimination (RFE) and Relief were performed for feature selections. Classifications were performed using 10 ML classifiers. Results were evaluated using a leave one out cross-validation analysis.

**Results:**

We compared the AUC of all pipelines on the validation dataset with FeAture Explorer (FAE) software. The pipeline using a PCC dimension reduction, relief feature selection, and gaussian process (GP) classifier yielded the highest area under the curve (AUC) using 15 features. When the “one-standard error” rule was used, FAE also produced a simpler model with 13 features, including S(5,-5)SumAverg, S(3,0)InvDfMom, Skewness, WavEnHL_s-3, Horzl_GlevNonU, Horzl_RLNonUni, 135dr_GlevNonU, WavEnLL_s-3, Teta4, Teta2, S(5,5)DifVarnc, Perc.01%, and WavEnLH_s-2. The AUCs of the training/validation/test datasets were 1.000/0.965/0.979, and the accuracies, sensitivities, and specificities were 0.890, 0.880, and 0.920, respectively. The best algorithm was GP whose AUCs of the training/validation/test datasets by the two-dimensional reduction methods and four feature selection methods were greater than approximately 0.800. Especially, the AUCs of different datasets were greater than approximately 0.900 using the PCC, RFE/Relief, and GP algorithms.

**Conclusions:**

We demonstrated the feasibility of combining artificial intelligence and the radiomics from Fs-T2WI to differentially diagnose SRCMTs and Non-SRCMTs. This non-invasive approach could be very promising in clinical oncology.

## Introduction

Malignant tumors in the nasal and paranasal sinuses are rare, comprise less than 1% of all malignancies and about 3% of head and neck malignancies (1, 2), including small round cell malignant tumors (SRCMTs) and non-SRCMTs. SRCMTs form a specific group of malignancies in the nasal and paranasal sinuses based on neuroectodermal, soft tissue, and hematopoietic differentiation, such as seen in rhabdomyosarcoma (RMS), malignant melanoma (MM), olfactory neuroblastoma (ONB), neuroendocrine carcinoma (NEC), and lymphoma. In contrast, non-SRCMTs form another common group of malignant tumors in the nasal and paranasal sinuses based on epithelial differentiation, including squamous cell carcinomas (SCCs) and adenoid cystic carcinomas (ACCs) (3). The distinction between these two groups is crucial as tumors are variably managed with radiation, chemotherapy, conservative medical therapy, local surgery, exenterative surgery, and multimodal therapy, indicating that therapeutic decisions, surgical planning, and prognoses are very different for each tumor type (4).

Conventional magnetic resonance imaging (MRI) has limitations of its own when differentiating between SRCMTs and Non-SRCMTs. Under the circumstances, as texture analysis (TA) techniques, by using mathematically defined features, can analyze pixel distributions, intensities and dependencies, it can provide a wealth of information beyond what can be seen with the human eye and thus can be used to characterize SRCMTs and Non-SRCMTs, quantitatively (5). Other sequences, such as the apparent diffusion coefficient, have been used to discriminate benign and malignant nasal and paranasal sinus lesions or different histopathologic types of sinonasal malignancies (6–11). However, less attention has been given to the application of TA for fat-suppressed T2-weighted MR images (Fs-T2WI) collected as part of routine clinical practice.

As a branch of artificial intelligence, machine learning (ML) includes various algorithms that can enhance diagnosis, treatments and follow-up results in neuro-oncology medicine by analyzing huge complex datasets (12, 13). More importantly, not depending on user experience, ML is more objective than other conventional analyses and has good repeatability. To our knowledge, no studies using TA and ML for differentiating sinonasal SRCMTs from non-SRCMTs have been reported. To bridge this gap, this retrospective study was intended to evaluate the potential value of the ML-based Fs-T2WI texture analysis for distinguishing SRCMTs from non-SRCMTs. To achieve the optimal predictive ability and clinical utility, we compared two-dimensional reduction, four feature selection methods and ten ML algorithms.

## Materials And Methods

### Patients

We used the surgical pathology database from January 1, 2018, to January 1, 2021, in our hospital. Exclusion criteria were (1) patients who received treatments before MRI scans and (2) inadequate image quality. All methods were performed in accordance with the relevant guidelines and regulations, and the informed consent requirement was waived. This retrospective study was approved by the Institutional Ethics Review Committee of our hospital.

### Image acquisition

Patients were examined with a 3T MR scanner (MAGNETOM Skyra, Siemens Healthcare, Erlangen, Germany) with a standard head coil. The MRI scan protocols included: axial fat-suppressed T2-weighted imaging (Fs-T2WI) (TR/TE= 5000/117 ms, matrix=256 x 256, field of view=24 x 24 cm^2^, slice thickness=5 mm, intersection gap =1mm).

### Extraction of Textural Features

MaZda software (version 4.7, The Technical University of Lodz, Institute of Electronics, http://www.eletel.p.lodz.pl/mazda/) was used for the analyses. We applied the limitation of dynamics to μ± 3δ(μ: mean grey-level value, δ: standard deviation) (14) to achieve reliable results for the MRI texture classifications. Regions of interest (ROIs) on the Fs-T2WI images of the largest layer were selected. Two physicians delineated ROIs manually along the edge of the lesion and filled the lesion in with a red marker, excluding the various necrotic and cystic regions. In total, 271 features were extracted for each ROI. The number of radiomics features based on feature classes were as follows and shown in **Table 1**: (i) 9 histogram features based on the number of pixel counts in the image that possessed a certain grey-level value (15); (ii) 220 grey-level co-occurrence matrix (GLCM) features based on the extraction of statistical information about the distribution of pixel pairs (16); (iii) 20 grey-level run-length matrix (GLRLM) features based on searching the image for runs that have the same grey-level values in a pre-defined direction (17); (iv) a 5 auto-regressive model (ARM) based on the weights associated with four neighboring pixels and the variance of the minimized prediction error; (v) 12 wavelet transform (WAV) features on texture frequency components extracted from the energies computed within the channels (18); and (vi) 5 absolute gradient statistics (AGS) features based on smooth or steep variations, resulting in low or high gradient values (15). Multiple GLRLMs were computed along the 0°, 45°, 90°, 135°, and z-axis directions, and 1, 2, 3, and 4 pixels. Multiple GLCMs were computed along four different angles (horizontal, vertical, diagonal 45°, and diagonal 135°).

**Table 1:** Texture analysis methods and the corresponding texture features.

Method	Texture feature parameters
Histogram(9)	mean, variance, skewness, kurtosis, and percentiles (1%, 10%, 50%, 90% and 99%)
Grey-level CO-occurrence matrix(GLCM)(220)	angular second moment(AngScMom), contrast, inversedifferent moment(IDM), entropy(Ent), correlation(Correlat), sum of squares(SumOfSqs), sum average(SumAverg), sum variance(SumVarnc), sum entropy(SumEntrp), difference variance(DifVarnc), difference entropy(DifEntrp) along the 0°, 45°, 90°, 135° and z‐axis directions and 1, 2, 3 and 4 pixels
Grey‐level run‐length matrix(GLRLM)(20)	run length nonuniformity(RLNonUni), grey level nonuniformity(GLevNonU), long run emphasis(LngREmph), short run emphasis(ShrtREmp), fraction of image in runs(Fraction) of four different angels (horization, vertical, digonal45, and digonal135)
Auto‐regressive model(ARM)(5)	Teta1, Teta2, Teta3, Teta4, Sigma
Wavelets transform(WAV)(12)	energy computed from the low–low frequency band within the first image scale(WavEnLL_s-1), WavEnLH_s-1, WavEnHL_s-1, WavEnHH_s-1, WavEnLL_s-2, WavEnLH_s-2, WavEnHL_s-2, WavEnHH_s-2, WavEnLL_s-3, WavEnLH_s-3, WavEnHL_s-3, WavEnHH_s-3
Absolute gradient statistics(AGS)(5)	absolute gradient mean(GrMean), variance(GrVariance), skewness(GrSkewness) kurtosis(GrKurtosis), nonzeros(GrNonZeros)

### Feature Selections

Computer-generated random datasets were used to assign 70% of datasets to the training set and 30% of the datasets to the independent test set. FeAture Explorer software (FAE, V 0.3.6) software on Python (3.7.6) (https://github.com/salan668/FAE) was used. Firstly, the synthetic minority oversampling technique (SMOTE) was used to balance the training dataset. This method works by taking each minority class sample and introducing synthetic examples along the line segments joining any/all of the k minority class nearest neighbors. The neighboring points were randomly chosen depending on the amount of over-sampling required. Secondly, we normalized the dataset by Z-score Normalization, which subtracts the mean value and divides the standard deviation for each feature. Lastly, we used a Pearson Correlation Coefficient (PCC) and principal component analysis (PCA) to reduce the dimensions. PCC is used for each pair of two features to reduce the row space dimensions of the feature matrix (19). If the PCC was larger than 0.99, one of them was randomly removed. PCA is an unsupervised feature reduction technique that explains the variance-covariance structure of a set of variables through linear combinations. Analysis of Variance (ANOVA) and Kruskal-Wallis (KW) and Recursive Feature Elimination (RFE) and Relief (20) were used for the feature selection. ANOVA was a common analytic method to explore the significant features corresponding to the labels. The KW is a non-parametric version of ANOVA, which hypothesizes that the population median of all groups is equal. The relief selects the sub-data set and finds the relative features according to label recursivity. The goal of the RFE is to select features based on a classifier by recursively considering a smaller set of features. The feature number range was set from 1 to 20.

### Classification Performances

The classification performances were tested using 10 ML algorithms, including the support vector machine (SVM), linear discriminant analysis (LDA); auto-encoder (AE); random forests (RF); linear regression (LR); logistic regression using Lasso (LRLasso); ada-boost (AB); decision tree (DT); gaussian process (GP); and naive Bayes (NB)(**Table 2**).

**Table 2:** The parameters of the algorithms.

Algorithms	Parameters
SVM	C=1.0, kernel='rbf', degree=3, gamma='scale', coef0=0.0, shrinking=True, probability=False, tol=0.001, cache_size=200, class_weight=None, verbose=False, max_iter=- 1, decision_function_shape='ovr', break_ties=False, random_state=None
AE	hidden_layer_sizes=(100), activation='relu', *, solver='adam', alpha=0.0001, batch_size='auto', learning_rate='constant', learning_rate_init=0.001, power_t=0.5, max_iter=200, shuffle=True, random_state=None, tol=0.0001, verbose=False, warm_start=False, momentum=0.9, nesterovs_momentum=True, early_stopping=False, validation_fraction=0.1, beta_1=0.9, beta_2=0.999, epsilon=1e-08, n_iter_no_change=10, max_fun=15000
LDA	solver='svd', shrinkage=None, priors=None, n_components=None, store_covariance=False, tol=0.0001
RF	n_estimators=100, *, criterion='gini', max_depth=None, min_samples_split=2, min_samples_leaf=1, min_weight_fraction_leaf=0.0, max_features='auto', max_leaf_nodes=None, min_impurity_decrease=0.0, min_impurity_split=None, bootstrap=True, oob_score=False, n_jobs=None, random_state=None, verbose=0, warm_start=False, class_weight=None, ccp_alpha=0.0, max_samples=None
LR	penalty='l2', *, dual=False, tol=0.0001, C=1.0, fit_intercept=True, intercept_scaling=1, class_weight=None, random_state=None, solver='lbfgs', max_iter=100, multi_class='auto', verbose=0, warm_start=False, n_jobs=None, l1_ratio=None
LRLasso	alpha=1.0, *, fit_intercept=True, normalize=False, precompute=False, copy_X=True, max_iter=1000, tol=0.0001, warm_start=False, positive=False, random_state=None, selection='cyclic'
AB	base_estimator=None, *, n_estimators=50, learning_rate=1.0, algorithm='SAMME.R', random_state=None
DT	criterion='gini', splitter='best', max_depth=None, min_samples_split=2, min_samples_leaf=1, min_weight_fraction_leaf=0.0, max_features=None, random_state=None, max_leaf_nodes=None, min_impurity_decrease=0.0, min_impurity_split=None, class_weight=None, ccp_alpha=0.0
GP	kernel=None, *, optimizer='fmin_l_bfgs_b', n_restarts_optimizer=0, max_iter_predict=100, warm_start=False, copy_X_train=True, random_state=None, multi_class='one_vs_rest', n_jobs=None
NB	alpha=1.0,binarize=0.0,fit_prior=True,class_prior=None

### Evaluations

The results were evaluated using leave-one-out cross-validation(LOOCV). Using LOOCV, learning sets were created by taking all samples but one, which was used as the validation set. The accuracy, sensitivity, and specificity were also calculated at a cutoff value that maximized the value of the Youden index. The area under the receiver operator characteristics curve (AUC) for the classification of results was calculated for each tested condition.

## Results

Of the 171 consecutive patients with a pathologic diagnosis of SRCMTs or Non-SRCMTs over a 2-year period from January 2018 until January 2021, seven were excluded for poor MRI image quality, and 164 patients were finally selected for the study. There were 70 patients with SRCMTs and 94 patients with Non-SRCMTs; RMS (n=16), lymphoma(n=18), MM (n=14), NEC (n=14), ONB (n=8), SCC (n=66), and ACC (n=28). There were 94 males and 70 females in the entire cohort. The mean age of the patients was 55.22 years with a range of 13 to 87 years. After removing invalid cases automatically with FAE, 162 cases were included with 68 SRCMTs and 94 Non-SRCMTs. We assigned 70% of the datasets to the training set (114 patients with 48 SRCMTs and 66 Non-SRCMTs) and 30% of datasets to the independent test set (48 patients with 20 SRCMTs and 28 Non-SRCMTs).

**Figure 1:**
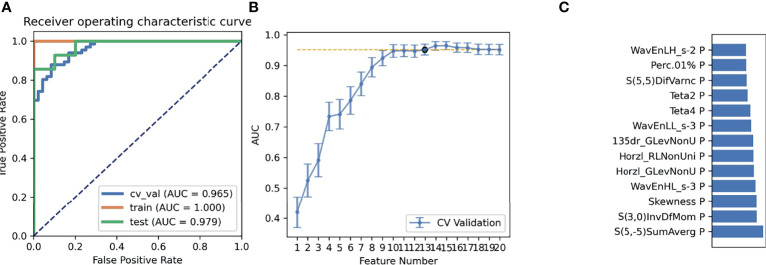
Performance of models generated using Pearson correlation coefficient (PCC) analyses, and Relief and Gaussian process (GP) algorithms. **(A)** Receiver operating characteristic (ROC) curves of this model on different datasets, **(B)** FeAture Explorer software suggested a candidate 13-feature model according to the “one-standard error” rule, and **(C)** A contribution of features in the final model.

**Figure 2:**
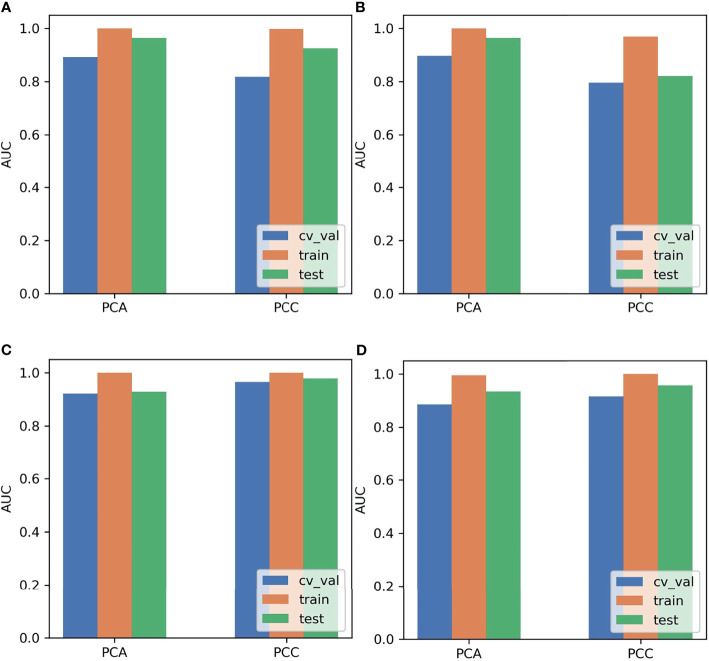
Areas under the curve (AUCs) of the different datasets using Pearson correlation coefficient (PCC) and principal component analysis (PCA) methods and Relief and Gaussian process (GP) algorithms. **(A)** Analysis of Variance (ANOVA), **(B)** Kruskal-Wallis (KW), **(C)** Relief, and **(D)** Recursive Feature Elimination (RFE).

**Figure 3:**
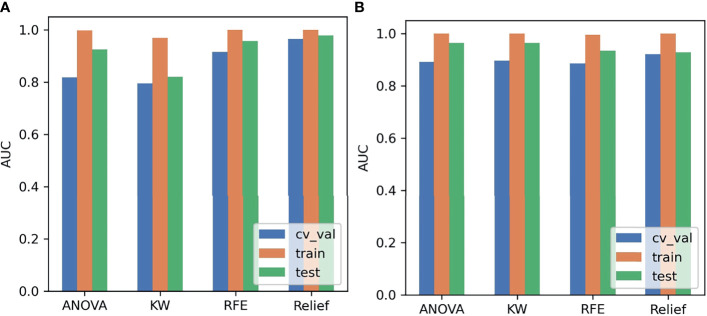
Areas under the curve (AUCs) on different datasets using the Analysis of Variance (ANOVA), Kruskal-Wallis (KW), and Recursive Feature Elimination (RFE) and Relief using Gaussian process (GP). **(A)** Pearson correlation coefficient (PCC), **(B)** principal component analysis (PCA).

**Figure 4:**
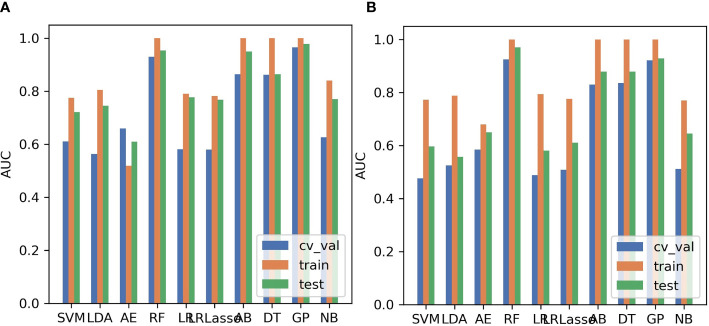
Areas under the curve (AUCs) of different datasets using 10 machine learning algorithms. **(A)** Pearson correlation coefficient (PCC), **(B)** principal component analysis (PCA). SVM, support vector machine; LDA, linear discriminant analysis; AE, auto-encoder; RF, random forests; LR, linear regression; LRLasso, logistic regression *via* Lasso; AB, ada-boost; DT, decision tree; GP, Gaussian process; NB, naive Bayes.

The SMOTE technique was used to automatically create 18 synthetic SRCMTs samples in the training set by operating in the feature space. We compared the AUC of all pipelines on the validation dataset with FAE. The pipeline using PCC dimension reduction, Relief feature selection, and a GP classifier yielded the highest AUC using 15 features. When the “one-standard error” rule was used, FAE also produced a simpler model with 13 features (21), whose ROC curves are shown in **Figure 1**. The AUCs of the training/validation/test datasets achieved 1.000/0.965/0.979, and the accuracy, sensitivity, and specificity were 0.89, 0.88, and 0.92 (**Supplementary Materials**), respectively. Features selected by FAE were S(5,-5)SumAverg, S(3,0)InvDfMom, Skewness, WavEnHL_s-3, Horzl_GlevNonU, Horzl_RLNonUni, 135dr_GlevNonU, WavEnLL_s-3, Teta4, Teta2, S(5,5)DifVarnc, Perc.01%, and WavEnLH_s-2 (the weight=1.48, 1.29, 1.28, 1.25, 1.21, 1.19, 1.19, 1.13, 1.10, 1.02, 1.00, 0.99, and 0.98). The AUCs of the training/validation/test datasets by the two-dimensional reduction methods and four feature selection methods were greater than ~0.800 using GP algorithm (**Figure 2**). Especially, the AUCs of different datasets were more than about 0.900 using the PCC, RFE/Relief, and GP algorithm (**Figures 3**, **4**).

## Discussion

This study investigated the potential value of the Fs-T2WI texture analysis of maximum tumor solid components for distinguishing SRCMTs from non-SRCMTs with ML. The key findings were as follows: (1) The pipeline using PCC dimension reduction, relief feature selection, and GP classifier yielded the highest AUC. (2) The best algorithm was GP whose AUCs of the training/validation/test datasets by the two-dimensional reduction methods and four feature selection methods were greater than approximately 0.800. Especially, the AUCs of different datasets were more than about 0.900 using the PCC, RFE/Relief, and GP algorithm. (3) TA with ML appears to be most helpful in tumor differentiation using standard Fs-T2WI routinely acquired with a high accuracy of 0.89.

Radiomics data contain first-, second-, and higher-order statistics (22). First-order statistics are described as the distribution of individual voxel values regardless of spatial relationships and are generally histogram-based features that refer to statistical parameters of pixel intensities within the ROI, such as mean, variance, skewness, kurtosis, and Perc.10%. Second-order statistics describe texture features. Specifically, they describe statistical interrelationships between voxels with similar or dissimilar contrast values, such as the co-occurrence matrix, which is calculated from the intensities of pixel pairing, with the spatial relationship of pixel pairing defined. Higher-order statistics impose filter grids on images to extract repetitive or nonrepetitive patterns, such as wavelets, which are data on texture frequency components extracted from the energies computed within the channels. In 2015, Fujima et al. (23) assessed the utility of the histogram analysis on tumor blood flow (TBF) obtained with pseudo-continuous arterial spin labeling to differentiate SCC and lymphoma in nasal or sinonasal cavities, achieving an accuracy of 0.87 for the mean TBF, the coefficient of variation, and kurtosis. In 2019, this group (24) also applied histograms and TAs for Fs-T2WI to differentiate SCC and lymphoma in the head and neck and found that the relative mean signal, contrast, and homogeneity could be useful. Another study (25) applied GLCM and WAV, using the ANOVA, rank-sum test, and SVM classification feature selections to diagnose osteosarcoma and achieved an accuracy of 0.82-0.96. Muramatsu et al. (26) applied GLCM and WAV using artificial neural network, SVM, and RF algorithms to classify and diagnose malignant and benign breast masses, achieving AUCs of 0.83-0.86. Finally, 440 radiomics features were applied, including histograms, GLCMs, GLRLMs, and WAVs, using 24 feature selection methods and three classification methods to predict lung cancer histologic subtypes (27)and they found that the Relief and NB algorithms achieved higher accuracies and the highest AUCs compared with other studied algorithms. Similarly, in our study, the Relief feature selection and GP classifier yielded the highest AUCs using the histogram, GLCM, GLRLM, and WAV. The Relief can avoid a heuristic search and select relevant features in linear time based on the given features and training instances regardless of the complexity of the target concept to be learned. The GP classification is a nonparametric method based on a Laplace approximation used for approximating the non-Gaussian posterior by a Gaussian method. It can easily handle a variety of problems, such as an insufficient capacity for the classical linear method, complex data types, and the curse of dimensions (28). Sovizi et al. reported that they achieved high sensitivity results using GP classification model (29). Consistent with their results, the AUCs of different datasets by GP algorithm were greater than approximately 0.900 with the RFE/Relief feature selection methods. RFE is recursively repeated in the pruned set, removing the least important features until the desired number of feature selections is eventually reached. Chatterjee et al. (30) have applied TAs to differentiate melanoma, dysplastic nevi, and basal cell carcinoma in dermoscopic images using SVM-RFE, achieving an accuracy of 0.952. Vamvakas et al. (31) applied SVM-RFE using 3D TA to classify low- and high-grade gliomas. With a LOOCV, they achieved an accuracy of 0.955 and an AUC of 0.955.

Some studies reported their diverse automated or semi-automated segmentation methods. For example, Muzzamil et al. proposed lung CT image segmentation using intensity thresholding (32). Husham et al. (33) and Malathi et al. (34), for another example, analysed between active contour and Otsu thresholding segmentation algorithms in segmenting brain tumor MRI and pleura diseases CT, respectively. In addition, Hussein et al. (35) proposed a new Viola–Jones model for the segmentation of ovarian and breast ultrasound images. Artificial neural networks and SVM have been tried for division of nasopharyngeal carcinoma, respectively (36, 37). The accuracy and consistency of the tumor delineation plays an important role in differential diagnosis. However, most of the tumors in the site of the nasal cavity and paranasal sinus adjacent to the air in Fs-T2 maps are without edema areas. Thus, we chose manual segmentation by experts not automated or semi-automatic segmentation to determine the boundary.

There were some limitations. First, as the SRCMTs studied were of various histologic types, subgroup analyses in more details should be performed in future studies after obtaining a larger sample size and a careful consideration of the study groups. Second, our model used manually delineated ROIs performed along the edge of the lesion. Segmenting precise tumor regions is the focus of future work. In our further studies, we will propose a multiparametric MRI investigation including ADC, T2-weighted MRI and dynamic contrast-enhanced MRI involving early and delayed phases to generate a robust model to differentially diagnose SRCMTs and Non-SRCMTs by segmenting precisely three-dimensional tumor regions in a larger sample.

## Conclusions

We demonstrated the feasibility of combining artificial intelligence and radiomics using Fs-T2WI in the differential diagnosis of SRCMTs and Non-SRCMTs. This approach could be a very promising non-invasive method in clinical oncology.

## Data Availability Statement

The original contributions presented in the study are included in the article/**Supplementary Material**. Further inquiries can be directed to the corresponding author.

## Ethics Statement

The studies involving human participants were reviewed and approved by the Institutional Ethics Review Committee of West China Hospital. Written informed consent from the participants’ legal guardian/next of kin was not required to participate in this study in accordance with the national legislation and the institutional requirements. Written informed consent was not obtained from the individual(s), nor the minor(s)’ legal guardian/next of kin, for the publication of any potentially identifiable images or data included in this article.

## Author Contributions

CC, YQ and JC initiated this study, participated in its design, performed study selection, data extraction, and data analysis. CC drafted the manuscript. FG supervised all aspects of the study. XZ revised the language. All authors contributed to the article and approved the submitted version.

## Funding

This study was supported by the National Natural Science Foundation of China (no. 81930046, 81771800, and 81829003).

## Conflict of Interest

Author XZ was employed by Siemens Healthineers Ltd., Shanghai, China.

The remaining authors declare that the research was conducted in the absence of any commercial or financial relationships that could be construed as a potential conflict of interest.

## Publisher’s Note

All claims expressed in this article are solely those of the authors and do not necessarily represent those of their affiliated organizations, or those of the publisher, the editors and the reviewers. Any product that may be evaluated in this article, or claim that may be made by its manufacturer, is not guaranteed or endorsed by the publisher.
